# Platelet-rich plasma regulating the repair of ultraviolet B–induced acute tissue inflammation: adjusting macrophage polarization through the activin receptor–follistatin system

**DOI:** 10.1080/21655979.2021.1944026

**Published:** 2021-06-30

**Authors:** Gajin Park, Wen Qian, Mei-Jie Zhang, Yi-He Chen, Li-Wen Ma, Ni Zeng, Qian Lu, Yue-Yue Li, Wei-Wei Ma, Xu-Feng Yin, Bing-Rong Zhou, Dan Luo

**Affiliations:** Department of Dermatology, the First Affiliated of Nanjing Medical University, Nanjing, China

**Keywords:** Platelet-rich plasma, UVB-induced acute photodamage, macrophage polarization, activin, inflammation

## Abstract

Ultraviolet B (UVB) is one of the most common exogenous factors in skin aging, especially photoaging. Once a large amount of UVB accumulates within a short period of time, skin tissue can become inflamed. It has also been found in clinics that platelet-rich plasma (PRP) can promote wound repair; therefore, the aim of this study was to identify the mechanism by which PRP repairs UVB-induced skin photodamage. We used PRP of Sprague-Dawley rats with the two-spin technique in the established acute UVB radiation photodamage model and harvested the corresponding skin after 1, 7, and 28 d. Hematoxylin and eosin staining was used to observe tissue inflammation. We found that PRP reduces inflammation in the early stages of UVB-induced acute skin damage, and then promotes the proliferation of collagen in the middle and late stages. Moreover, PRP can stimulate Act A and M1 polarization in the early stage, while inhibiting activin A (Act A) and inducing M2 polarization in the middle and late stages. In conclusion, this study demonstrates that PRP plays an important regulatory role in helping reduce UVB-induced acute skin tissue inflammation by adjusting macrophage polarization, which alleviates skin inflammation and stimulates collagen regeneration.

## Introduction

Sunburn is a complex process of inflammation that occurs after acute photodamage to the skin caused mainly by ultraviolet B (UVB). Excessive sun exposure or artificial UV radiation can cause sunburn, especially among people classified with the Fitzpatrick scale for skin types between III and IV. A National Health Interview Survey [1] conducted in 2015 on 31,162 nationally representative American adults found that 34% of participants had experienced at least one sunburn that year. Many proinflammatory factors and chemokines are involved in this process, which mediate different cellular responses at different stages [2]. In addition, research suggests that long-time exposure or long-term accumulation of UVB is the main cause of skin cancer [3].

As a member of the transforming growth factor beta (TGF-β) superfamily, activin A (Act A) reacts quickly in the early stages of skin inflammation, and is highly expressed in inflammatory tissues/cells [4], such as macrophages, monocytes, T cells, B cells, mast cells, and dendritic cells [5]. When Act A binds to an activin receptor (ACVR), it is activated and functions through the intracellular Smad2/3 signaling pathways. Once follistatin (FST) irreversibly binds to Act A [6], the combination of Act A and ACVR and activation of smad2/3 pathways are inhibited. ACVR IIA is the main type of ACVR [7]; therefore, research suggests that Act A can dynamically regulate acute inflammation through the ACVR IIA–FST system, stopping additional inflammation and inhibiting existing inflammation [6]. Macrophages work at nearly all stages of wound healing, although they play different roles in different stages of inflammation. Because of different macrophage polarizations within different inflammation environments, macrophages regulate the balance between inflammation and repair [8].

Platelet-derived growth factor has been found to significantly increase the expression of Act A, and platelet-rich plasma (PRP), which contains a large number of growth factors, chemokines, and cytokines, has be proposed to promote Act A expression; however, the specific mechanism by which this action is implemented has not been studied [9]. Clinical observations have found that PRP can accelerate wound healing and promote tissue proliferation and regeneration after trauma [10]. Karina also confirmed that the combination of stromal vascular fraction (SVF) and PRP accelerated the healing of burn wounds in SD rats [11]. Studies have also shown that PRP significantly improves skin elasticity [12] by promoting collagen proliferation and skin rejuvenation. At the same time, in the treatment of striae distensae (or ‘stretch marks’), PRP injections were found to increase the number of local elastic fibers and induce their morphological improvement after 3 months [13]. To facilitate the detection to evaluate PRP potency, platelet counts are often used in clinics, which is best when the platelet concentration in PRP reaches six times higher than original blood; however, the specific mechanism by which PRP repairs tissue inflammation has not been identified, especially in the research on UVB-induced acute skin photodamage.

In this study, we observed the effect of PRP on inflammation after acute skin photodamage in rats. The mechanisms by which PRP reduced and repaired skin inflammation induced by UVB and whether PRP regulates the process of UVB-induced acute skin tissue inflammation by adjusting macrophage polarization through the ACVR IIA-FST system were analyzed.

## Materials and methods

### PRP preparation

Eight female Sprague-Dawley (SD) rats 56–62 d old were purchased from Beijing Weitonglihua Laboratory Animal Technology Co. Ltd. and reared in a specific-pathogen-free laboratory before the study.

**Figure 1.**
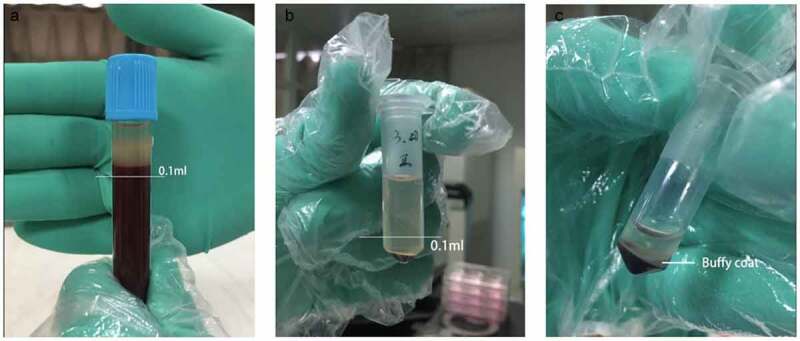
Separation of blood cell after secondary centrifugation a. After the first centrifugation, the supernatant and upper 0.1 ml blood cells were collected. b. After the second centrifugation, the lower 0.1 ml supernatant and cells below were collected. c. The middle part full of platelet is called Buffy Coat. What we used were the whole Buffy coat with the least plasma and red blood cell

An intraperitoneal injection of 0.5 mL/100 g 10% chloral hydrate was administered to anesthetize the rats, after which 4 mL blood was sampled from the abdominal aorta into a 5-mL Eppendorf (EP) tube that had been wet with 3.8% sodium citrate. At a ratio of 1:9, 3.8% sodium citrate was added to the blood and the solution gently mixed. After an air embolism as induced to sacrifice the rats and after suturing the incision, the rats were returned to the Animal Care Facility of Nanjing Medical University. Using the two-spin technique, we centrifuged the blood at 18°C at specific speeds and times depending on the learned literatures. After the first centrifugal force at 300 × g for 5 min, the blood samples were stratified. The supernatant and upper 0.1 mL blood cells were collected into a new 0.5-mL EP tube (Figure 1(a)). After the second centrifugal force at 700 × g for 17 min, we retained only the lower 0.1 mL supernatant and the cells below it (Figure 1(b,c)), mixed the blood with equal amounts of phosphate-buffered saline, and incubated the solution in a shaking bed at room temperature for 30 min. The prepared PRP was stored in the refrigerator at 4°C until use.

Platelets were counted to calculate and record the changes in platelet concentration.

### UVB radiation and treatments

**Figure 2.**
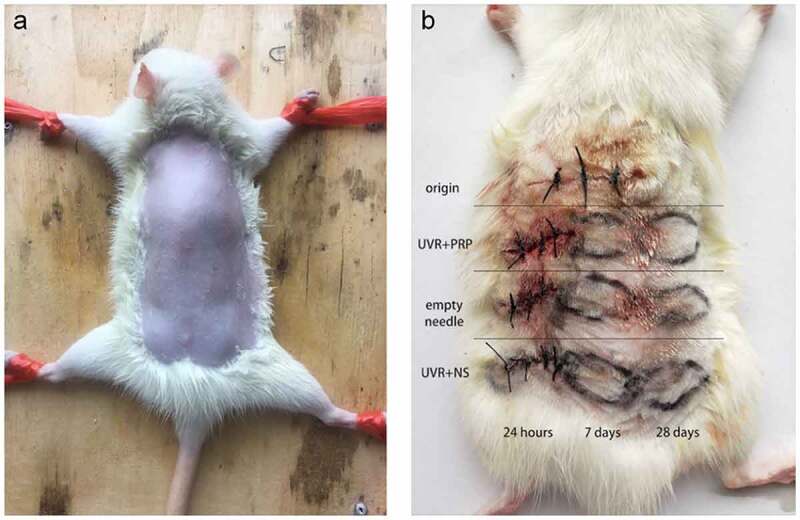
Preparation of rat back skin and different treatments a. After anesthesia, the back skin of the rats was carefully shaved to form an exposure area of 4 cm ×5 cm in size. b. Rule of nines was used to divide the exposed area of back skin for different treatments. Samples (about 10 mm long x 3 mm wide x 2 mm thick) were taken at appropriate time in different groups

After preparing the skin (Figure 2(a)), the back skin of each rat was divided into 10 regions. One sample was taken near the neck before radiation and marked as the origin group to indicate the initial level of SD rats. After one-time irradiation with 1000 mJ/cm^2^, an intradermal injection of either PRP or normal saline (NS) (Figure 2(b)) was administered into a 1-cm adjacent area once a day for 7 d. The treated skin was harvested after 24 h, 7 d, and 28 d, and marked as either the UVR+PRP or UVR+NS group. The animals were sacrificed using the air embolism technique. All samples were analyzed using hematoxylin and eosin (HE) staining, Masson staining, quantitative reverse transcription polymerase chain reaction (qRT-PCR), and Western blotting.

### HE staining

After fixing with 10% formaldehyde solution, the samples were pretreated using standard protocols and stained using a Hematoxylin-Eosin Staining Kit (Keygentec, Nanjing, China) according to the manufacturer’s protocols.

### Masson staining

After fixing with 10% formaldehyde solution, the samples were pretreated using standard protocols and stained with hematoxylin (Jiancheng, Nanjing, China) for 5 min, washed with distilled water for 1 min, and differentiated using 0.1% HCl for 1 min to wash back to blue. The samples were then stained using acid fuchsin (Yike, Guangzhou, China) solution for 8 min and washed with distilled water for 30 s, after which 1% phosphomolybdic acid solution (Yike, Guangzhou, China) was applied for 5 min, solid green (Yike, Guangzhou, China) applied for 3 min after drying, and tap water used to rinse the samples. The samples were dewaxed and sealed to observe the proliferation of collagen in the skin tissue.

### qRT-PCR

Total RNA was isolated using the TRIzol reagent (Invitrogen) and reverse transcribed into cDNA using the Thermo RT Kit (Thermo Fisher Scientific, USA) according to the manufacturer’s protocols. The mRNA expression levels of ACVR IIA, FST, TNF-α, IL-1β, 1 L-12b, Arg-1, IL-10, Dectin-1, collagen 1a1, and collagen 3a1, normalized to β-actin, were detected using the SYBR Green Master Mix Kit (Roche, Basel, Switzerland) according to the manufacturer’s protocols. The RNA primers were shown in Table 1. Each RNA level was quantified using the 2^−∆∆Ct^ method. The experiment was conducted in triplicate and repeated at least three times, so at least 6 times in total.

**Table 1.** The details of RNA primers

gene	forward primer	reverse primer	amplified bands size
**β-actin**	5-GCAGGAGTACGATGAGTCCG-3	5-ACGCAGCTCAGTAACAGTCC −3	74
**ACVR IIA**	5-GGCTTCTCGTTGTACTGCTG-3	5-CCTGCGTGTTTCTGCCAATA-3	159
**FST**	5-TGTAAAGAGCAGCCGGAACT −3	5-GGCAGGCACTGGAGTAAGTC-3	208
**TNF-α**	5-CATCCGTTCTCTACCCAGCC-3	5-CCCAGAGCCACAATTCCCTT-3	125
**IL-1β**	5-GACTTCACCATGGAACCCGT −3	5-GGAGACTGCCCATTCTCGAC-3	104
**1 L-12b**	5-TTCTCCCTCAAGTTCTTCGTCC-3	5-AACGCACCTTTCTGGTTACACT-3	86
**Arg-1**	5-AGCTGGGAATTGGCAAAGTG-3	5-AACTCAGGTGAATGGGCCTT-3	78
**IL-10**	5-CCAGCTGGACAACATACTGC-3	5-TTCTGGGCCATGGTTCTCTG-3	136
**Dectin-1**	5-AGCGTGATTTGCTCAGTGTC-3	5-CCTCATCCCTTCACGTCTCA-3	86
**Collagen 1a1**	5-GAGAGGTGAACAAGGTCCCG-3	5-AAACCTCTCTCGCCTCTTGC-3	153
**Collagen 3a1**	5-CCTGCAGGAAAGGATGGAGA-3	5-ATACCAGCTGGGCCTTTGAT-3	92

### Western blotting

Protein was extracted from the tissue samples and separated using sodium dodecyl sulfate-polyacrylamide gel electrophoresis (SDS-PAGE). The protein was then blotted onto an Immobilon-P membrane (Millipore, Billerica, MA, USA), which was then blocked in secondary antibodies (Keygentec) of goat anti-rabbit enzyme horseradish peroxidase (HRP) (1:2000 dilution) and goat anti-mouse HRP (1:3000 dilution). Antibody binding was visualized using BeyoECL Plus reagents (Keygentec). The details about the antibodies are provided in Table 2. The protein bands were semiquantified using the Bio-Rad UV Transilluminator (Hercules, CA, USA).

**Table 2.** Changes of platelet count in different rat blood samples before and after the preparation

Number	Platelet count before centrifugation	Platelet count after centrifugation	Multiple of concentration rise
1	478 × 10^9	2527 × 10^9	5.29
2	555 × 10^9	3089 × 10^9	5.57
3	320 × 10^9	2293 × 10^9	7.17
4 5 6 7 8	532 × 10^9 671 × 10^9 421 × 10^9 497 × 10^9 632 × 10^9	2145 × 10^9 3804 × 10^9 2534 × 10^9 3185 × 10^9 3785 × 10^9	5.05 5.67 6.02 6.41 5.99

### Statistical analyses

The date is presented as the mean ± standard deviation (SD). The Student’s *t*-test was used to analyze the results between two groups while one-way analysis of variance (ANOVAs) was used to analyze the results among multiple groups. SPSS 13.0 (SPSS Inc., Chicago, IL, USA) was used to statistically analyze all the experimental data. P < 0.05 was considered statistically significant.

## Results

### Preparing and verifying PRP

After 5 mL blood was collected from each rat, 1 mL PRP was obtained using the two-spin technique. Eight SD rats produced 8 mL PRP in total. The increase in platelet concentration in the plasma before and after preparation was calculated (Table 2), and the concentrations rose by 5- to 7-fold.

### PRP strengthens inflammation 7 d after treatment and increases collagen fibers by 28 d after treatment

**Figure 3.**
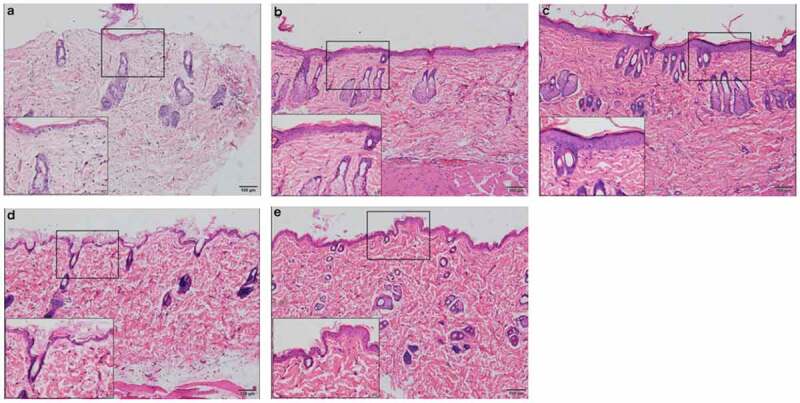
Rat skin histopathological changes at different points with HE staining (100 fold and 400 fold) a. rat skin histopathological before irradiation. b. 7 days after UVB irradiation in NS treatment group. c. 7 days after UVB irradiation in PRP treatment group. d. 28 days after UVB irradiation in NS treatment group. e. 28 days after UVB irradiation in PRP treatment group

Seven days after UVR treatment (Figure 3 (b-c)), the epidermis was thicker than that in the initial state (Figure 3(a)), which was characterized by an increase in epidermal cells accompanied by cell vacuoles and edema, an increase in the number of hair follicles and sebaceous glands, and a few inflammatory cells scattered throughout the dermis. At the same time, inflammation in the PRP group was stronger than that in the NS group.

**Figure 4.**
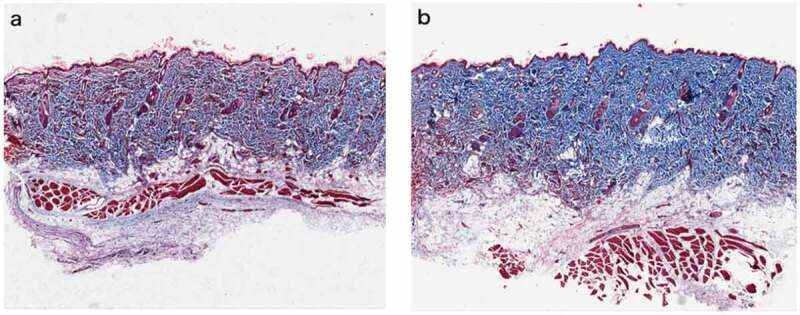
Rat skin histopathological changes of collagen with Masson staining (100 fold) Masson staining was used to observe collagen fibers (blue stained part was collagen fibers) a. Rat skin in NS group on the 28th day. b. Rat skin in PRP group on the 28th day

At 28 d after UVR treatment (Figure(3d-e)), inflammation was reduced compared to that 7 d after treatment. We observed that the thickness of epidermis and the number and morphology of the epidermis cells basically returned to their initial states, and the dermis became thicker. Compared with those in the initial state, dermatoglyphics had deepened, and the epidermis and dermis were slightly thickened. There were more collagen fibers in the PRP treatment group than in the NS treatment group, and they were arranged more closely together and orderly (Figure 4).

**Figure 5.**
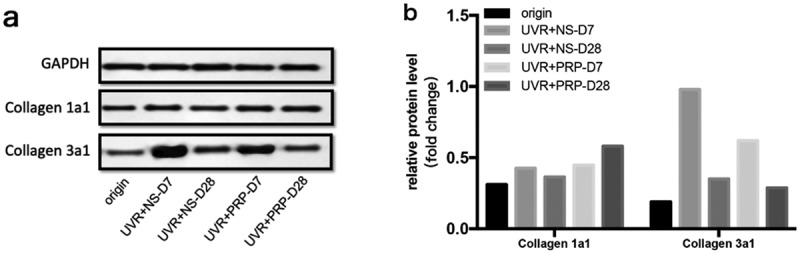
Protein expression changes of Collagen 1a1 and Collagen 3a1 (Western blotting) a. Western analysis of Collagen 1a1 and Collagen 3a1 at different time points of NS and PRP group in rat skin. b. Quantitative protein levels of Collagen 1a1 and Collagen 3a1 expression in the two group at different time points

At the protein level, in general, the expression of collagen 1a1 in both the NS and PRP groups slowly and smoothly increased after UVR treatment. The expression of collagen 1a1 in the RP group 28 d after treatment was higher than that in the NS group (P < 0.01). The expression of collagen 3a1 increased 7 d after treatment but decreased 28 d after treatment, and the expression of collagen 3a1 in NS group was higher than that in PRP group (P < 0.01) 7 d after treatment (Figure 5).

**Figure 6.**
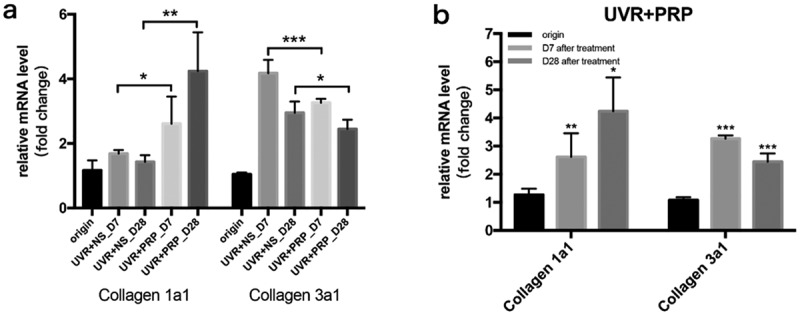
mRNA expression changes of Collagen 1a1 and Collagen 3a1 (qRT-PCR) a. The expression of Collagen 1a1 and Collagen 3a1 mRNA at different points and statistical analysis between the two groups. *p < 0.05, **p < 0.01 and ***p < 0.001. b. The expression of Collagen 1a1 and Collagen 3a1 mRNA at different points and statistical analysis in PRP group. *p < 0.05, **p < 0.01 and ***p < 0.001 *vs*. origin group

At the mRNA level, the expression of collagen 1a1 increased with time after UVR treatment, especially in the PRP group, which showed higher levels than in the NS group (P < 0.05); these levels were highest 28 d after treatment in the PRP group. The expression of collagen 3a1 increased 7 d after treatment but then decreased 28 d after treatment, particularly in the PRP group (P < 0.001). The expression of collagen 3a1 in the PRP group was lower than that in NS group at the same point in time (P < 0.05), and then was highest in the NS group 7 d after treatment (Figure 6).

### Activin activity increased in the early stage while inhibited in the middle and late stages

**Figure 7.**
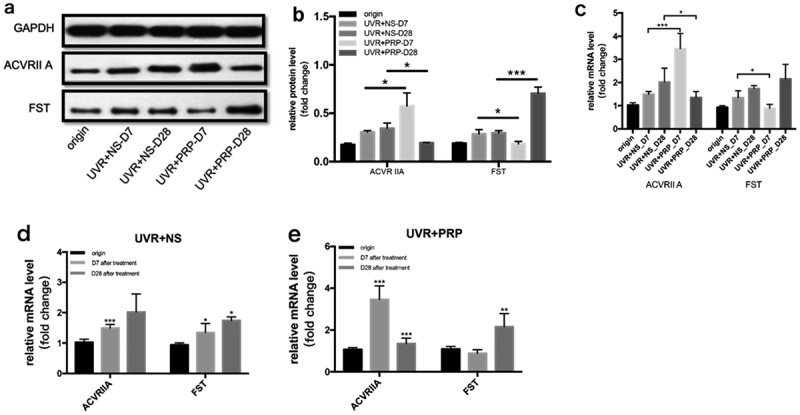
Changes of ACVR IIA and FST expression in rat skin at 7 and 28 days in NS and PRP treated group. a. Representative images of western blots of ACVR IIA and FST protein in NS and PRP treated group at different time points. b. Quantitative protein levels of ACVR IIA and FST are shown. c. The expression of ACVR IIA and FST mRNA at different points and statistical analysis between groups. *p < 0.05 and ***p < 0.001. d,e. The expression of ACVR IIA and FST mRNA at different points and statistical analysis in groups. *p < 0.05, **p < 0.01 and ***p < 0.001 *vs*. origin group

At the protein level, the expression of ACVR IIA was higher in the PRP group than in the NS group 7 d after UVR treatment (P < 0.05). FST expression was highest 28 d after treatment in the PRP (P < 0.001) (Figure 7(a-b)).

At the mRNA level, the expression of ACVR IIA was highest in the PRP group 7 d after UVR treatment, and FST expression was highest in the PRP group 28 d after UVR treatment; however, the expression of ACVR IIA and FST in the NS group increased with time. The expression of ACVR IIA increased in the PRP group 7 d after treatment (P < 0.001) and was higher than that in the NS group (P < 0.001), but then decreased in the PRP group 28 d after treatment (P < 0.001) and was then lower than that in the NS group (P < 0.05). The expression of FST in PRP group was lower than that in NS group 7 d after treatment (P < 0.05) and significantly increased in PRP group 28 d after treatment (P < 0.001) (Figure 7(c-e)).

### PRP can stimulate M1 polarization in the early stage

**Figure 8.**
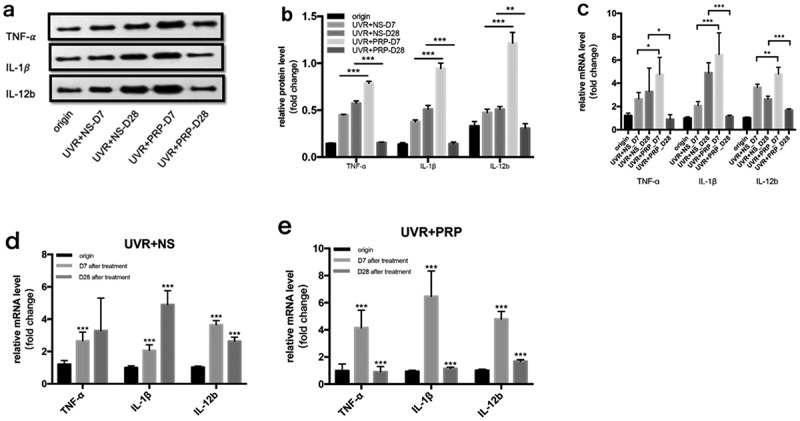
Changes of TNF-α, IL-1γ and IL-12b expression in rat skin at 7 and 28 days in NS and PRP treated group. a. Representative images of western blots of TNF-α, IL-1β and IL-12b protein in NS and PRP treated group at different time points. b. Representative images of western blots of TNF-α, IL-1βand IL-12b protein in NS and PRP treated group at different time points. c. The expression of TNF-α, IL-1β and IL-12b mRNA at different points and statistical analysis between groups. *p < 0.05, **p < 0.01 and ***p < 0.001. d,e. The expression of TNF-α,IL-1γ and IL-12b mRNA at different points and statistical analysis in groups. ***p < 0.001 *vs*. origin group

At the protein level, the expressions of TNF-α, IL-1β, and IL-12b in the NS group increased 7 and 28 d after UVR treatment, while the expressions in the PRP group increased 7 d after treatment but then decreased at 28 d. In addition, the expression in the PRP group was significantly higher 7 d after treatment (P < 0.001) but lower at 28 d (P < 0.01) that that in the NS group during those same time periods (Figure 8(a-b)). At the mRNA level, the expressions of TNF-α, IL-1β, and IL-12b in the NS group 7 d after UVR treatment were higher than those before treatment (P < 0.001). At 28 d after treatment, TNF-α levels showed no significant change while IL-1β increased and IL-12b significantly decreased (P < 0.001). In the PRP group, the expressions of these three 7 d after UVR treatment significantly increased (P < 0.001) and were higher than those in the NS group (P < 0.05); however, these levels then decreased 28 d after treatment (P < 0.001) and were lower than those in the NS group (P < 0.05) (Figure 8 (c-e)).

### PRP can induce M2 polarization in the middle and late stages

**Figure 9.**
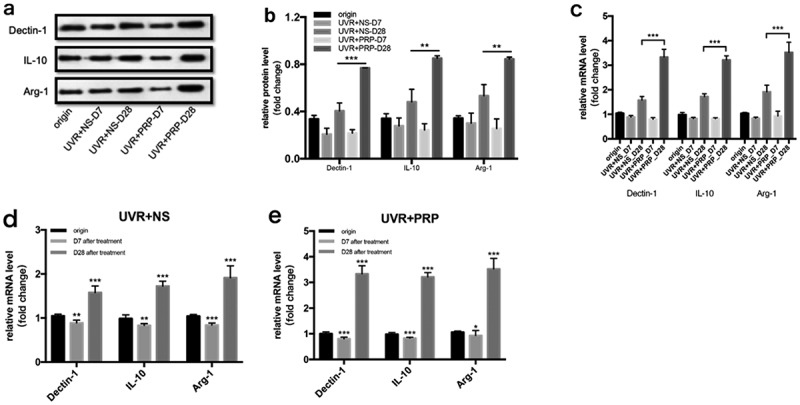
Changes of Dectin-1, IL-10 and Arg-1 expression in rat skin at 7 and 28 days in NS and PRP treated group. a. Representative images of western blots of Dectin-1, IL-10 and Arg-1 protein in NS and PRP treated group at different time points. b. Quantitative protein levels of Dectin-1, IL-10 and Arg-1 are shown. c. The expression of Dectin-1, IL-10 and Arg-1 mRNA at different points and statistical analysis between groups. **p < 0.01 and ***p < 0.001. d,e. The expression of Dectin-1, IL-10 and Arg-1 mRNA at different points and statistical analysis in groups. *p < 0.05, **p < 0.01 and ***p < 0.001 *vs*. origin group

At the protein level, the expressions of Dectin-1, IL-10, and Arg-1 decreased 7 d and increased 28 d after UVR treatment in both the NS and PRP groups. The expression levels of these three in the PRP group were higher than those in the NS group (P < 0.01) (Figure 9 (a-b)).

At the mRNA level, the expression changes in Dectin-1, IL-10, and Arg-1 showed a tendency similar to those in the Western blot results, but with a more significant difference (P < 0.001) (Figure 9 (c-e)).

## 4 Discussion

Sunlight is a necessary external factor in our daily lives; however, excessive exposure, especially to UVB, can cause acute skin photodamage from sunburn and solar dermatitis. These reactions are characterized by the release of active materials, epidermal cell necrosis, dermal vasodilation, tissue edema, and accelerated melanin synthesis, and epidermal edema, necrosis, and pigmentation will appear gradually. PRP is more and more widely used in clinical work, and its indications continue to expand. Applications and efficacies of PRP in dermatology comprise facial rejuvenation, reductions in scars and vitiligo, and the synergistic effect of CO_2_ fractional laser therapy [10]; however, there is no research about PRP therapy for acute photodamage. With its abundance of platelets, growth factors, chemokines, and cytokines, PRP can accelerate skin healing, proliferation, and regeneration. In this study, we used the two-spin technique to prepare PRP [14], which increased our final platelet concentration in PRP by 5- to 7-fold.

Using a rat model with the skin photodamaged after UVR treatment, we observed skin inflammation 7 d after treatment. We evaluated this inflammation using epidermis thickness and edema of epidermis cells and found that it was higher in the PRP group than in the NS group; however, at 28 d after UVB irradiation, the skin integrity and collagen density in the PRP group were better than those in the NS group. Meanwhile, the results of Masson staining 28 d after treatment further indicated that the quantity, density, and uniformity of collagen in the PRP group were significantly better than those in the NS group.

Considering the intradermal injection, acupuncture could also partially stimulate collagen proliferation [15], the collagen also increased in NS group in the late stage. In any case, the increase in collagen 1a1 was more significant in the PRP group, while that of collagen 3a1 was more obvious in the NS group, which indicated that PRP helps the skin reach the stage of inflammation and enter the remodeling stage much earlier than it would without PRP. After degradation of collagen 3a1 and synthesis of collage 1a1 [16], PRP can promote skin repair and even play a role in skin rejuvenation.

Collagen has long been defined as an indicator of skin aging [17]. Normal adult dermis contains mainly collagen 1a1 (85–90%) and collagen 3a1 (10–15%), which link to skin elasticity and toughness, respectively. Collagen 1a1 is the most important factor in skin elasticity and its decline leads to skin aging [18]. Collagen 3a1 is associated with scar formation and its reduction can inhibit scar formation [19].

Our preliminary results of PRP treatment suggest that PRP functions in regulating inflammation and repairing acute photodamage. In another words, PRP strengthens the inflammatory response in the early stages of damage and promotes tissue repair and remodeling in the middle and late stages. These specific mechanisms are discussed below.

Immune cells are believed to have phenotypic variability and functional diversity in immune responses, being activated into different subtypes within specific environments. They have characteristic distribution and surface factors, and can secrete corresponding cytokines to play their respective roles [20]. Such polarization has also been found in a mononuclear phagocytosis system.

Two main macrophage polarizations have been recognized – classical activation (M1 macrophages) and alternative activation (M2 macrophages) [21]. M1 macrophages polarize with the high expression of proinflammatory cytokines, such as IL-1, IL-12, and TNF-α, promoting an inflammatory response. Although polarization tends to be M2 macrophages with a high expression of Arg-1 and Dectin-1, an anti-inflammatory cytokine IL-10 [22,23] is produced to participate in pro-proliferation and regeneration.

Our research found that 7 d after UVR treatment, M1 macrophage–related molecules were overexpressed, while M2 macrophage–related molecules were under-expressed in the PRP group to promote inflammation. The opposite results were observed 28 d after treatment in the PRP group (i.e., a high expression of M2 macrophage–related molecules and a low expression of M1 macrophage–related molecules) to inhibit inflammation and promote repair. Another related issue assumes the Arg-1^+^ M2 macrophages are reparative, necessary for collagen production, and thus profibrotic [24]. Meanwhile, stimulation of the wounded skin by Dectin-1 is observed to increase keratinocyte proliferation and the re-epithelialization of human skin [25]. The high expression of Arg-1 and Dectin-1 in M2 macrophages could be the main reason for tissue repair in the middle and late stages.

Act A also shows a complex function of switching between proinflammation and anti-inflammation in other research [4]; therefore, we talk further about how Act A plays an exact regulatory role in complex inflammation and whether it regulates inflammation by influencing macrophage polarization.

When the expression levels of ACVR IIA and FST were simultaneously detected in our study, the ACVR IIA–FST system that regulates activin activity showed obvious temporal characteristics. Our results suggest that PRP can induce a high expression of ACVR IIA and a low expression of FST in the early stages of acute photodamage, increasing the activity of Act A, while in the middle and late stages, under-expressed ACVR IIA and overexpressed FST were induced to inhibit the activity of Act A. In accordance with the results of HE staining, PRP enhanced the inflammatory response 7 d after treatment and skin repair 28 d after treatment. The result of the ACVR IIA–FST system is consistent with the tendency of macrophages to polarize.

In recent years, more and more studies have shown a correlation between Act A and macrophage polarization, but the specific roles of both are controversial. Although a study published in *Journal of Immunology* has indicated that Act A induces macrophage expression of Arg-1 (overexpression in M2 macrophages) and inhibits the expression of iNOS induced by IFN-γ (overexpression in M1 macrophages), which suggests that Act A can induce the alternative activation of macrophages [26]. Another study published in Blood has shown that Act A can activate proinflammatory phenotypes and inhibit the acquisition of anti-inflammatory markers, inducing the polarization of M1 macrophages to play a proinflammatory role [27]. Although the relationship between Act A and macrophage polarization is complex, our results show that Act A has the same tendency as M1 macrophage in acute photodamage and indicate that Act A induces M1 macrophages to promote inflammation.

In addition, several studies have suggested that aberrant M2 macrophages induce macrophage involvement in tumor development and that the progression and TNF-α inhibitors might provoke the potential risk of oncogenesis [28]. TNF, which is overexpressed in our experiment in the early stage and then under-expressed, is believed to be the major anti-M2 pathway because the depletion of TNF will cause a relative increase in M2 gene expression when the conditions are favorable for the M2 pathway [29]. Unfortunately, the duration of our study was limited to 28 d after UVR treatment and the TNF-α expression was still high at that time. With long-term monitoring of TNF-α and other tumor-associated markers, we suggest that the mechanism by which M2 macrophages affect tumor development and progression and the efficacy of PRP in skin cancer can be explored.

However, what we have done remains limitations. A larger experimental animal sample is needed for the more accurate efficacy evaluation. To verify the mechanisms preliminarily explored in this experiment, additional cellular-level studies are suggested to be conducted to elucidate this potential mechanism, including the knockdown and overexpression of ACVR IIA and FST. Meanwhile, PRP is one product with multiple components like growth factors, which ones play more critical role in this progress remains unclear.

## 5 Conclusions

We preliminarily found that PRP affects activin activity through the ACVR IIA–FST system and induces specific macrophage polarization during different stages of inflammation, thereby participating in the inflammation and repair of acute skin photodamage. This finding provides new ideas about clinical treatments of acute photodamage and scientific theoretical basis for the application of PRP.
